# Accelerated Atherosclerosis

**DOI:** 10.3390/medicina62101898

**Published:** 2026-09-30

**Authors:** Alexandru-Cristian Nechita, Dan-Cristian Popescu, Mara Diaconu, Adrian-Claudiu Popa, Bogdan Marcu, Vasilica Enache

**Affiliations:** 1 Department of Cardiology, Clinical Emergency Hospital Sfântul Pantelimon, 021659 Bucharest, Romania; cardionechita@yahoo.com (A.-C.N.); popescu_dan_95@yahoo.com (D.-C.P.); mara2228@gmail.com (M.D.); adrian_popa_mailbox@yahoo.com (A.-C.P.); vasi.enache@eucardios.ro (V.E.); 2 Faculty of Medicine, Carol Davila University of Medicine and Pharmacy, 020021 Bucharest, Romania; 3 Faculty of Medicine, Transilvania University, 500036 Brasov, Romania

**Keywords:** accelerated atherosclerosis, systemic lupus erythematosus, rheumatoid arthritis, genetic factors, ultrasonography, therapeutic interventions

## Abstract

Accelerated atherosclerosis (AA) denotes a disproportionately early and rapid progression of arterial atheroma unpredictable by standard tools. Clinicians often rely on simplified frameworks for cardiovascular risk assessment, which may overlook genetic and pathological conditions associated with AA. Some of these are well established, others are considered future therapy targets. This review examines the conditions in which AA is most firmly established, including rheumatoid arthritis (RA), systemic lupus erythematosus (SLE), antiphospholipid syndrome, chronic kidney disease (CKD), human immunodeficiency virus (HIV), diabetes mellitus, and psoriasis. We also examined the importance of genetic determinants of AA, with particular emphasis on clonal hematopoiesis of indeterminate potential (CHIP) and lipoprotein (a) (Lp(a)); contemporary imaging-based detection and monitoring; the diagnostic role of circulating non-coding microRNAs; and complementary protein biomarkers of residual inflammatory risk. After identifying biomarkers of increased cardiovascular risk and possible AA, clinicians should assess atherosclerotic disease directly using the available imaging techniques, most of which are non-invasive. The clinical consequences of AA are discussed and a comprehensive review of the therapeutic options is provided to offer a practical approach.

## 1. Introduction

AA is a clinical condition associated with various diagnostic and therapeutic challenges. There is no universally accepted definition, although several criteria have been proposed. AA is the opposite of spontaneous atherosclerosis; while the latter needs decades to achieve a significant degree of vascular stenosis, AA goes to the same result over a short period of time—from a few months up to 2–5 years. Another recently developed term is the rapid progression of atherosclerosis. The clinician should be able to identify promptly the conditions prone to promoting the rapid progression of plaques and provide the patient with a comprehensive panel of imaging and functional investigations. These investigations can establish the stage of disease progression and the extent of vascular involvement. The earlier the condition is identified, the more effective the intervention is likely to be. Providing patients with measures that minimise residual cardiovascular risk is essential.

Intense and persistent inflammation can accelerate atherosclerotic progression, but it acts alongside—not instead of—causal exposure to apoB-containing lipoproteins and other conventional risk factors. Potential triggers of sustained inflammation include autoimmune diseases, clinical interventions, metabolic and genetic factors, and newer exposures such as immune checkpoint inhibitor therapy. Retention of apoB-containing lipoproteins within the arterial intima is central to atherogenesis; endothelial dysfunction and inflammation amplify plaque initiation and progression rather than replacing lipid exposure as the initiating mechanism.

To tackle the challenge posed by this form of atherosclerosis, the first step is identification of the process, using all the imaging available techniques. We mention in the forefront of this diagnostic armamentarium ultrasound of the peripheral arteries and CT-derived angiography imaging of the main systemic arteries and of the coronary arteries. Invasive coronary angiography should be reserved for established clinical indications, particularly when revascularization is being considered; it should not be used as a routine monitoring tool for suspected AA.

## 2. Definition

All too often, we tend to forget that arteries are complex organs with at least three major types of tissues, namely, endothelial, connective, and smooth muscle, that interact to create the functional structure. The contribution of vascular innervation adds further complexity. We also tend to simplify the perception regarding blood vessels, considering them unitary structures acting the same in different territories. Each vascular bed reflects a finely regulated network of local and systemic interactions.

Arterial beds may respond differently due to genetic factors that translate into specific phenotypes and to environmental factors. Classic cardiovascular risk factors such as being male or smoking, in addition to diabetes, hypertension, and low-density lipoprotein (LDL) cholesterol, are just a part of the picture in the complex process of plaque formation and progression.

Atherosclerosis is a lipid-driven, immunoinflammatory disease of the arterial intima. The qualifier “accelerated” is applied when atheroma develops earlier, progresses faster, and produces clinical events at a higher rate than baseline cardiovascular risk models predict for a given age, sex, and lipid profile [1].

The concept is operationalized in two complementary ways: epidemiologically, as a standardized event rate exceeding that of a matched control; and mechanistically, as the amplification of conventional atherogenesis by chronic systemic inflammation, endothelial activation, immune-cell recruitment, and dysfunctional lipoprotein handling [1,2]. A defining feature is that established equations systematically underestimate risk—contemporary risk equations such as PREVENT and SCORE2 fail to capture disease-specific mediators—so that “accelerated” is, in practice, synonymous with “under-predicted by standard tools” [2,3].

The coronary computed tomography angiography (CCTA)-based PARADIGM study proposed a definition of rapid plaque progression: percent atheroma volume (PAV) increase of more than 1% per year. In these patients, metabolic syndrome predicted rapid progression [4].

Importantly, AA is not merely a faster form of ordinary atheroma. Serial imaging studies show that the same traditional risk factors carry greater atherogenic weight in the inflammatory milieu; for instance, LDL isolated from patients with active SLE is more atherogenic to endothelial cells than LDL from the same patients in remission, and the “lipid paradox” of RA—low total, LDL, and high-density lipoprotein (HDL) cholesterol coexisting with high event rates—reflects inflammation-driven changes in cholesterol turnover and HDL function rather than a benign lipid profile [1].

## 3. Evolution of Terminology

Early or rapidly progressive atherosclerosis has been recognized since the 1950s, initially mainly through post-mortem examinations. With the subsequent development of sophisticated non-invasive imaging, clinicians became able to quantify disease onset and monitor its rate of progression. Contemporary usage increasingly distinguishes intimal atherosclerosis from medial arteriosclerosis (arterial stiffening and Mönckeberg-type medial calcification), which are mechanistically distinct and especially relevant in CKD; conflating them obscures both pathophysiology and therapeutic targets [5].

The most recent conceptual reframing recasts AA as one manifestation of “residual inflammatory risk”—the fraction of cardiovascular risk that persists after LDL cholesterol is lowered to guideline targets—and situates it within an “immune–metabolic–vascular” axis in which persistent immune activation reshapes the vascular microenvironment toward endothelial dysfunction, maladaptive myeloid activation, and immunothrombosis [6]. This shift in language has direct clinical consequences, because it reframes inflammation from an epiphenomenon into a measurable, modifiable therapeutic target [6,7].

## 4. Conditions Associated with Accelerated Atherosclerosis

The traditional risk factors contribute across vascular territories, although their relative associations and plaque phenotypes can differ between arterial beds. Immune mechanisms and plaque evolution differ among vascular territories, resulting in distinct rates of progression and complications. For example, femoral lesions may appear earlier in the life course but progress more slowly, be more calcified, and be associated with fewer atherothrombotic events. Researchers identified major differences between the vascular beds regarding smooth muscle cell (SMC) characteristics, inflammatory cells and perivascular adipose tissue [8]. Particular attention has been given to conditions that promote atherosclerosis as a consequence of their (auto)inflammatory nature (Table 1).

RA is an independent cardiovascular risk factor recognised by European guidelines, with cardiovascular disease risk substantially increased compared with the general population. Systemic inflammation, seropositivity, long disease duration, extra-articular manifestations, and high disease activity are each associated with faster subclinical atheroma progression, and carotid intima–media thickness (CIMT), together with markers such as homocysteine and asymmetric dimethylarginine, correlate with risk. Despite this excess risk, RA is systematically under-screened and under-treated for cardiovascular risk factors [1].

In SLE, cardiovascular disease accounts for approximately one-quarter of deaths, and the relative risk of coronary events in women aged 35–44 years has been reported as being as high as fiftyfold above background [2]. In a 7-year prospective study of 115 patients with SLE matched 1:1 to controls, atherosclerotic plaque developed substantially more often in patients with SLE, and progression was governed by both traditional and disease-specific factors [9]. A head-to-head comparison of SLE and RA found that in SLE, independent determinants of subclinical progression included antiphospholipid antibodies (odds ratio [OR] 2.00, *p* = 0.043) and the SCORE index (OR 2.87, *p* = 0.019), and—among patients maintaining low disease activity—cumulative corticosteroid dose (OR 1.38, *p* = 0.013) and disease duration (OR 1.20, *p* = 0.022), underscoring glucocorticoid exposure as a modifiable accelerant [10]. In antiphospholipid syndrome (APS), carotid and femoral plaque burden is equivalent to that of diabetes, and roughly half of APS patients develop a new plaque over 7 years, with antiphospholipid antibody positivity independently linked to endothelial dysfunction and thrombo-inflammatory atherogenesis [11,12].

CKD combines conventional atherogenesis with uremia-specific drivers—hyperphosphatemia, mineral-bone disorder, and loss of calcification inhibitors—producing both intimal atheroma and medial calcification [5]. Cardiovascular event rates rise steeply as glomerular filtration falls, and coronary artery calcium (CAC) progresses far faster than in the general population. In the Chronic Renal Insufficiency Cohort (CRIC), progressive CAC (an increase ≥50 Agatston units per year) independently predicted atherosclerotic events, heart failure, and all-cause mortality [13]. Advanced CKD is characterised by near-ubiquitous medial calcification, reported in roughly 80% of stage 5 patients, which is an independent predictor of events and is mechanistically distinct from atheroma [5,14].

People with HIV have excess cardiovascular risk due to residual immune activation and chronic inflammation superimposed on traditional risk factors; standard risk charts frequently underestimate this risk. The REPRIEVE trial confirmed that this risk is modifiable and that a substantial fraction of the benefit of statin therapy in this population exceeds what LDL reduction alone would predict, which is consistent with an anti-inflammatory contribution [15].

Hyperglycemia and metabolic dysregulation accelerate atheroma through oxidative stress, reduced nitric-oxide bioavailability, endothelial dysfunction, and inflammatory activation. In asymptomatic type 2 diabetes without known coronary disease, baseline pericoronary adipose tissue attenuation (PCATa)—a computed-tomography surrogate of coronary inflammation—predicted 12-month increases in total and non-calcified plaque volume (NCPV) and burden, linking a measurable inflammatory signal to prospective plaque growth [16].

Psoriasis is now regarded as a systemic inflammatory disorder associated with coronary microvascular dysfunction, high-risk plaque, vascular inflammation, and arterial stiffness, and it is frequently underestimated by clinical risk scores [17,18]. These conditions share a common template—immune-mediated endothelial activation driving accelerated atheroma—that also encompasses psoriatic arthritis and inflammatory bowel disease [17].

Cardiac allograft vasculopathy (CAV) is a transplant-specific form of AA driven by a complex interaction of alloimmune injury, innate immune activation, endothelial dysfunction, and conventional cardiovascular risk factors. It is characterized by diffuse, concentric intimal thickening that can involve both epicardial and intramyocardial vessels [19]. In this setting, intravascular ultrasound (IVUS) can detect early intimal disease beyond the sensitivity of angiography, while optical coherence tomography (OCT) provides high-resolution characterization of early intimal and plaque changes and can support serial assessment [20]. Importantly, invasive surveillance for CAV follows dedicated heart-transplant protocols and should not be extrapolated to routine invasive screening of patients with suspected AA from other causes.

Acute and post-acute COVID-19 have been associated with inflammatory, thrombotic, myocardial, and perivascular abnormalities. Observational imaging studies and mechanistic hypotheses have raised the possibility that these changes could interact with pre-existing atherosclerosis [21]. More recently, serial CCTA data have shown that prior SARS-CoV-2 infection is associated with faster progression of total and non-calcified coronary plaque burden, increased development of high-risk plaque features, and greater pericoronary inflammatory activity [22]. However, the available evidence remains observational and does not establish that post-COVID inflammation directly causes rapidly progressive atherosclerosis.

## 5. Genetic Factors

Genetic determinants are estimated to account for approximately 30–60% of susceptibility to atherosclerosis. Genetic and epigenetic factors interacting with the environment shape a phenotype (proteome, inflammation, lipid signature, blood pressure, immune system activation, coagulation problems, etc.), which leads to great variability regarding atherosclerosis onset and progression.

Genetic and epigenetic findings should be interpreted as modifiers of susceptibility, age at onset, plaque burden, or rate of progression, rather than as a separate mechanism that automatically defines AA. Their relevance to AA is strongest when they help explain atherosclerotic disease that appears earlier or progresses faster than expected by conventional risk assessment.

Familial hypercholesterolemia (FH) is caused by autosomal-dominant alleles that lead to defective LDL-R, proprotein convertase subtilisin/kexin type 9 (PCSK9), or apolipoprotein B (APOB), subsequently determining LDL-C elevation from birth and an enormous risk for coronary artery disease (CAD). Homozygous patients can present CAD from childhood, whereas heterozygous patients may develop such disease beginning in their 30s [23]. On average, in FH, PAV doubles for every 75 mmol/L of LDL x years of exposure [24]. Homozygous FH and heterozygous FH therefore represent well-established examples of markedly premature atherosclerotic disease driven by lifelong LDL-C exposure; however, the main emphasis of this review is on accelerated or disproportionate atherosclerosis that cannot be explained by extreme LDL-C elevation alone [25].

Lp(a) is a strongly heritable (70–90% genetically determined) LDL-like particle and an independent, causal risk factor for atherosclerotic disease and calcific aortic valve disease, affecting an estimated 1.5 billion people (18% of the general population) and 30–36% of the CAD population [26,27] with levels ≥50 mg/dL (≥125 nmol/L) [28,29]. In a multi-ethnic pooled US cohort, higher Lp(a) predicted long-term atherosclerotic events across sex and race/ethnicity [28], and in 9944 patients stratified by guideline risk, the adjusted hazard ratio for events in the highest Lp(a) group (≥75 mg/dL) versus the lowest (<30 mg/dL) was 1.75 (95% CI 1.25–2.46), rising to 2.18 (1.32–3.58) in very-high-risk patients [30]. Contemporary guidance now recommends measuring Lp(a) at least once for every adult [28]. Long-term data also link elevated Lp(a) to accelerated coronary plaque progression, low-density plaque, and pericoronary inflammation in serial imaging [31]. Familial elevation of Lp(a) is an inherited condition associated with premature atherosclerosis. From this class, a small number of responsible genes and agents (PCSK9, Angiopoietin-like 3 modulation (ANGPTL3), Apolipoprotein C-III (APOC3), triglyceride-rich lipoproteins and Lp(a)) carry the heavy burden for the atherosclerotic process. Other genes, although associated with lower impact, were also identified: CDKN2A/2B, SORT1, and CXCL12.

CHIP comprises somatic mutations arising in hematopoietic stem and progenitor cells. Its frequency increases with age and it is usually associated with mutation in epigenetic regulators such as DNMT3A, HDAC, and TET2 and genes coding receptors or proteins involved in signalling pathways [32]. It is an independent risk factor for myocardial infarction (MI), and is mediated by dysregulation of innate immunity, increased cytokine production, and inflammasome activation. TET2 (with a prevalence of 5–10% in individuals older than 65 years [33]) and JAK2-mutant CHIP are causally associated with larger atherosclerotic plaques through NLRP3 inflammasome activation and downstream interleukin (IL)-1β and IL-6 signalling; in a mechanistic and clinical link, an exploratory CANTOS analysis showed that carriers of TET2 CHIP derived greater major-adverse-cardiovascular-event reduction from IL-1β neutralisation. Experimentally, TET2 loss enhances not only atheroma but also atrial fibrillation incidence via the same inflammasome axis, illustrating the pleiotropy of these clones [34].

Epigenetic modifications add a further regulatory layer to transcription and translation without altering the underlying DNA sequence. The process can act directly on the chromatin fibre through DNA methylation and histone acetylation, without disrupting the genetic code per se, but locking the ability of effector enzymes to reach the DNA strand in a usable fashion. Or it can act in a more subtle and complicated pattern, on mRNA disruption. Epigenetic modifications are the bridge that connects environmental or metabolic cues to genetic programming and regulation of phenotype [35].

Aberrant DNA methylation was encountered in atherosclerotic plaques. Hypermethylation of anti-inflammatory genes leads to increased inflammation, whereas extensive hypomethylation can also lead to expression of pro-inflammatory genes, the final result being plaque formation and evolution. The methylation of the promoter of ATP-Binding Cassette (ABC) Transporter A1 diminishes macrophage cholesterol efflux, promoting its transformation into a foam cell [36].

Histone modifications do not affect the nucleotides in the DNA strand, but, through reversible methylation, acetylation and phosphorylation of histones, produce their effects. Enzymatic regulators such as HDAC and histone acetyltransferases (HATs) modify histone structure and thus allow or deny access of effectors to the nucleotides in the DNA strand [37]. Studies have shown reduction of NF-κB-linked inflammation response in endothelial cells. Histone methylation at H3K4me3 or H3K27me3 leads to macrophage transformation and plaque evolution. It influences smooth muscle cell phenotype, thereby affecting plaque composition and stability [38,39].

Most atherosclerotic cardiovascular disease (ASCVD) genetic risk is polygenic: highly distributed architecture containing thousands of common alleles, each with small effect, sum to compose an individual fingerprint. Recent genome-wide associative studies (GWAS) have identified potential loci for CAD and have presented a network of links with phenotype traits, leading to an associative image of effector genes and their translation status and effector pathways [40,41].

Due to the complexity of the envisioned genetic implications, in the last decade, genetic study of atherosclerosis has evolved from locus discovery to multi-variable mechanism-oriented approaches. Large international consortia have conducted GWAS, fine-mapped candidate loci and expanded evidence across non-European populations. These studies, combined with the latest functional genomic investigations (such as vascular expression quantitative trait locus (QTL) mapping and epigenomic annotation) have demonstrated a causal link with potential effector genes [42].

The information regarding the immense number of loci and their associative relations requires high-performance computational methods. Systematic stratification is therefore required to translate large genomic datasets into plausible molecular mechanisms and clinically relevant applications.

Tissue-relevant regulatory maps and vascular QTL provide information about the transcription levels of various gene alleles and about the splicing processes in specific vascular territories, thereby supporting the prioritization of candidate effector loci. One limitation is that available datasets often overrepresent endothelial cells [41,42,43].

Other new techniques, such as single-nucleus profiling and single-cell RNA sequencing, applied in atherosclerotic lesions, have provided valuable data regarding in situ endothelial phenotypes, macrophage states, inflammatory transcriptional status, and SMC transformation to fibroblast-like or osteochondrogenic phenotype.

These studies have also shown that many CAD-associated variants lie within non-coding regions, implicating regulatory mechanisms. There are situations where the functional regulator loci are not near, leading to long-range enhancer–promoter interactions.

High PRS for CAD offers clues outside the classic risk factors and is associated with higher lifetime risk and earlier disease onset [40]. PRS may shed light on which patients could benefit more from early and intensive therapy. They may also improve stratification of enrolment cohorts in clinical trials [44].

From a clinical perspective, the principal value of polygenic risk scores (PRS) may lie in identifying patients who require early, intensive LDL-C lowering or preventive treatment, despite borderline risk according to conventional models. Further research is warranted to establish for PRS a place within the patient workup in preventive cardiology [23,45].

Taken together, FH, Lp(a), CHIP, polygenic susceptibility, and epigenetic regulation illustrate different ways in which inherited or acquired biological factors can shift the expected timing or rate of atherosclerosis. For most polygenic and epigenetic markers, however, the evidence currently supports risk refinement and mechanistic understanding rather than an AA-specific diagnostic test.

## 6. Detection and Monitoring

Because standard equations under-predict risk in these populations, subclinical atherosclerosis imaging is central to detection. Vascular ultrasound of the carotid and femoral arteries quantifies CIMT and plaque and has been used to demonstrate accelerated progression in SLE and APS over multi-year follow-up [9,10]. CAC scoring stratifies risk and, in CKD, serial CAC captures the rapid calcification characteristic of that population; CAC progression adds prognostic information beyond baseline scores [13].

In the last 15 years carotid ultrasound became widely available, together with significant technological improvements. It has therefore become an important tool for cardiovascular risk assessment. From this examination the clinician receives two important sets of data, CIMT and carotid plaque dimensions and total burden. Whereas CIMT is more closely associated with cardiovascular risk factors, plaque burden more directly reflects atherosclerotic disease [46].

Carotid and femoral ultrasonography are among the most accessible methods for detecting atherosclerosis. This approach can be performed at the same time as cardiac ultrasound, increasing the diagnostic yield and prognostication value. In a population of about 500 subjects aged 36.2 years, and with low cardiovascular risk, after 12.8 years of follow up, premature atherosclerosis was found in 31.9% of the subjects. This was mostly independent of the traditional risk factors for ASCVD [47]. In a different cohort of 1569 patients, mean age 55 years and without documented ASCVD, subclinical atherosclerosis was found in 54.7%. After 10 years of follow-up, the cardiovascular event rate was 5.7%. Only patients with three or more plaques, compared with those without plaque, had a significantly higher event rate (HR 2.90; 95% CI 1.48–5.65; *p* = 0.002) [48]. The low event rate in this cohort is also explained by the very high prescription rates of statins and antiplatelet therapy, especially in those with several plaques. An important body of evidence supporting the monitoring of carotid artery progression was obtained from the clinical trials that followed the effect of statins on carotid atherosclerosis. Moreover, a two-year study from China showed a threefold slower rate of atherosclerosis progression in those treated with rosuvastatin, compared with placebo [49].

Carotid CIMT is an accessible parameter measurable by carotid ultrasound; for the presence of significant CAD, a value of over CIMT ≥ 1.0 mm has a sensitivity of 77%, positive predictive value of 82%, and negative predictive value of 65%, with carotid plaque presence having a sensitivity of 80% and specificity of 67%. The results suggest that carotid CCTA does not outperform carotid ultrasound [50].

According to the current classification, we can distinguish the following grades: Grade zero—no plaque, IMT < 1.5 mm; Grade I—Protuberant focal thickening of the vessel wall, IMT < 1.5 mm; Grade II—protuberant or diffuse vessel wall thickening, and CIMT > 1.5 mm, IMT 1.5–2.4 mm; Grade III—Protuberant or diffuse, IMT > 2.5 mm [51].

Carotid plaque area is a simple and reproducible measurement. In a series of 1686 patients divided into quartiles according to plaque area, those in the highest quartile had a 19.5% five-year risk of the composite outcome of stroke, MI, or cardiovascular death. The study also reported progression in 63%, regression in 28.2%, and no change in 16.2%, with corresponding outcome rates of 15.7%, 9.4%, and 7.6% (mc6), respectively [52]. Other authors suggested that total plaque volume change is a better predictor for major adverse cardiovascular events (MACE) at three years, compared to plaque area or IMT change [53]. A carotid atheroma volume below 0.09 mL on three-dimensional ultrasonography had a negative predictive value of 93.3% and a sensitivity of 98%, compared with 75% for two-dimensional plaque thickness below 1.35 mm [54]. Follow-up of known carotid stenosis should be guided by stenosis severity, symptoms, and dedicated vascular guidelines.

Intravascular imaging can quantify the progression of coronary atherosclerosis and assess the effects of therapies, as demonstrated in the REVERSAL, ASTEROID, and SATURN trials of high-intensity statin treatment [55,56,57]. After a period of about two years of therapy, the process of atherosclerosis progression was halted in approximatively 66% of patients on high doses of statins, with no statistical difference between atorvastatin 80 mg and rosuvastatin 40 mg/day. The pooled results of all the studies concerning this subject confirmed the validity of this strategy in a meta-analysis of 32 studies that included almost 5000 patients. The results suggest that achievement of an LDL < 80 mg/dL and HDL > 45 mg/dL are strongly associated with atherosclerosis regression expressed by a significant change in total atheroma volume (TAV) and also PAV [58]. Adding ezetimibe to standard statin therapy can also increase the rate of plaque regression [59]. Further reductions and a larger percentage of responders were obtained when adding PCSK9 inhibitors to the standard therapy. This strategy is useful for patients with statin intolerance [60,61]. Most of these IVUS studies enrolled conventional CAD populations rather than patients defined by AA. A clearer AA-specific application is cardiac allograft vasculopathy, in which IVUS and OCT can detect and characterize accelerated intimal disease after heart transplantation [19,20].

Serial CCTA has quantified plaque progression in research studies and selected clinical scenarios, but routine scheduled CCTA solely to monitor suspected AA is not guideline-established and should be weighed against radiation exposure, iodinated contrast, cost, and whether the result is likely to alter management [45]. When serial CCTA is performed for a justified indication or research protocol, acquisition and reconstruction methods should be standardized. TAV in mm^3^ and NCPV in mm^3^ should be used as quantifiers. Plaque progression should be considered if an increase in TAV > 10–20 mm^3^ or NCPV > 5–10 mm^3^ per year is identified. The advent and general implementation of AI-empowered CT machines have shortened the examination time and increased the accuracy of the results and reproducibility [62].

CCTA now enables tissue-level characterisation. PCATa—the mean CT density of perivascular fat, quantified between −190 and −30 Hounsfield units (HU)—is an investigational imaging marker associated with coronary inflammation. Observational studies have linked higher PCATa with adverse cardiovascular outcomes, but its roles in routine AA risk stratification or serial monitoring have not been established. In asymptomatic type 2 diabetes, baseline PCATa independently predicted 12-month growth in total plaque volume (β = 0.004, *p* = 0.02) and non-calcified plaque burden (β = 1.8, *p* = 0.02) after adjustment for age, sex, C-reactive protein, triglycerides (TG), and statin use, supporting PCATa as a research marker for dynamic coronary inflammation [16]. High-risk plaque features—low-attenuation non-calcified plaque, positive remodelling, and lipid-rich necrotic core—further refine detection and are modifiable by therapy in inflammatory disease [17].

A series of 1769 patients were followed for 4.7 years at baseline risk factors, and CCTA was performed looking for coronary calcium, degree of stenosis and low-attenuation plaque burden. Low-attenuation plaque burden was the strongest predictor of subsequent MI, with values above 4% associated with a fivefold increase in risk [63]. A CAC score of 0 is associated with a favourable prognosis in both diabetic and non-diabetic patients, although risk increases more rapidly over time in diabetes. For the highest CAC > 400, compared with CAC = 0, the hazard ratio was 4.64 in non-diabetic patients (95% confidence interval = 3.74–5.76) and 3.41 in diabetic patients (95% confidence interval = 2.22–5.22) [64]. CCTA patients who displayed plaque progression also had a higher incidence of cardiovascular events 27% vs. 0.3%, *p*< 0.001 [65].

In immune-mediated inflammatory diseases such as RA, psoriasis and SLE, CCTA may play a role in detection and monitoring of coronary plaques. In RA, up to 65% of patients show coronary calcium, and this is proportional with the duration of the disease; also, younger patients have disproportionate atherosclerosis, and there is more trivascular disease and more severe stenosis [66]. Biological medications and statins seem to decrease the rate of progression by decreasing both the number of new lesions and calcification in the old ones [67,68]. In psoriasis with or without arthritis, there is a higher prevalence than in control studies of both calcified and non-calcified plaques, in addition to more three-vessel and high-risk plaques [69,70]. SLE is associated with the highest burden of fibrotic plaques; non-calcified plaque was associated with higher disease activity and no traditional risk factors [71,72,73]. After a 3-year follow-up period in SLE patients there was recorded a rate of atherosclerosis progression of 18%, compared with 12.9% in control subjects [74]. These observational and serial-imaging data support CCTA in selected clinical or research settings but do not establish routine repeated CCTA for all asymptomatic patients with inflammatory disease.

## 7. Non-Coding MicroRNA in the Diagnosis of Accelerated Atherosclerosis

Noncoding RNAs (microRNA, long noncoding RNA) represent important regulatory tools for transcription and translation, leading to a fine genetic tuning. To date, noncoding RNAs have been identified as playing an important role in the atherosclerotic process: these molecules affect all cellular functions, participating in all processes that drive atherosclerosis [75,76]. At present, these molecules should be regarded as mechanistic and investigational biomarkers rather than validated tests for routine diagnosis of AA.

MicroRNAs are small RNA oligonucleotides that specifically bind the target mRNA and, upon establishing the bond, recruit the RISC complex (RNA-induced silencing complex), which leads to gene silencing and suppression of protein production. A specific miRNA can bind more than one mRNA, producing a pleiotropic translational effect [75].

In endothelial cells, a high-miR-126 environment increases the pro-angiogenic pathway, with a decrease in leukocyte adhesion being normally present in endothelial injury [77]. The miR-29a promotes inflammatory response and impairs the endothelial cell function. The miR-21 leads to apoptosis of endothelial cells [78]. Other miRNAs act on lipid metabolism. The miR-33a/b reduces macrophage cholesterol efflux through ABCA1 and ABCG1 inhibition, leading to foam cell formation [79]. MiR-122 and miR-148a influence systemic LDL levels through modifications in LDL-R expression [80,81]. Inflammatory pathways are not shielded from miRNA action. Some have anti-inflammatory effects: miR-124 restricts macrophage polarization [82] and miR-146a limits cytokine production linked to NF-κB signalling. Other miRNAs promote inflammation (miR-155 leads to activation of macrophage, miR-145/143 cluster downregulation promotes vascular smooth muscle cell proliferation and migration, whereas its upregulation promotes the contractile phenotype) [83,84]. In the intimal tissue, miR-29 family exponents inhibit the production of extracellular matrix, thus weakening the fibrous cap of the atherosclerotic plaque [85].

Long noncoding RNAs participate in the regulation of signalling, inflammation and metabolic processes in all blood-vessel cells. CDKN2B-AS1, in conjunction with 9p21 CAD locus, modulates inflammatory processes, as well as proliferation or apoptosis [86].

LINC00493, lincRNA-Cox2, and MALAT1 are also involved in inflammation response as well as in proliferation [87,88]. Long noncoding RNAs can participate in lipid homeostasis; LeXis and MeXis modulate hepatic and macrophage cholesterol metabolism [89], and this influences endothelial proliferation, angiogenesis, and inflammation. SENCR and SMILR act on vascular smooth muscle cells, changing the equilibrium between contractile phenotype and proliferation [90].

Circulating and extracellular-vesicle miR-155 also tracks plaque activity—decreasing during regression and increasing during progression—supporting a role in monitoring as well as diagnosis. In a case–control study of coronary heart disease and periodontal status, salivary miR-155 discriminated disease groups, with AUC (area under the curve) ranging from 0.69 to 0.99 (sensitivity 76.7–100%, specificity 53.3–96.7%), and also had discriminative power between coronary heart disease and periodontitis, with an AUC ≥0.84 [91]. Current limitations include the heterogeneity of pre-analytical methods, variable normalization, and the fact that most miRNAs are not disease-specific; neither single miRNAs nor multi-miRNA panels are currently validated for routine diagnosis or monitoring of AA.

## 8. Other Biomarkers

High-sensitivity C-reactive protein (hsCRP): Among statin-treated patients, residual inflammation indexed by hsCRP predicts recurrent events at least as strongly as LDL cholesterol. A collaborative analysis of three randomized trials found hsCRP to be a stronger predictor of MI, stroke, and cardiovascular death than LDL [7], and this was confirmed among 13,970 statin-intolerant patients in CLEAR Outcomes, where inflammation predicted events at least as strongly as cholesterol [92]. The 2025 American College of Cardiology scientific statement formalized residual inflammatory risk as an actionable target, noting that patients with an hsCRP >3 mg/L carry markedly higher event rates even when LDL is aggressively controlled [6].

Interleukin-6 (IL-6) is the central upstream cytokine of the CHIP–inflammasome axis and is being studied as both a biomarker and therapeutic target in CAD, CKD, autoimmune disease, and HIV [6,93]. Growth differentiation factor-15 (GDF-15), a stress- and inflammation-induced cytokine, is an independent predictor of mortality and disease progression across cardiovascular, renal, and metabolic disease, with interpretation dependent on the assay, threshold, and study population [94]. In APS, plasma GDF-15 above a 1200 pg/mL cut-off was independently associated with subclinical atherosclerosis (CIMT and carotid/femoral plaque) in 80 patients versus 40 controls, supporting further investigation as a risk marker in inflammatory conditions, where standard scores may underperform [11]. Disease-specific proteins—including sclerostin in SLE and vitamin K-dependent proteins predicting valvular calcification and mortality in haemodialysis—extend the biomarker repertoire for population-tailored risk assessment [95,96]. IL-6 and GDF-15 are not currently validated as routine AA-specific risk-stratification tests.

## 9. Clinical Outcomes

The clinical signature of AA is an excess of hard events—MI, ischemic stroke, and cardiovascular death—occurring at younger ages and higher rates than predicted. In SLE, cardiovascular disease is a leading cause of late mortality (≈25% of deaths), and plaque progression continues even when traditional risk factors meet European targets, indicating that conventional control is necessary but insufficient [2,9]. In CKD, CAC progression translates directly into excess atherosclerotic events, heart failure, and death [13], and event rates escalate as glomerular filtration declines [14]. Elevated Lp(a) confers durable long-term risk, with hazard ratios up to 2.18 in very-high-risk subgroups and evidence of accelerated plaque progression on serial CCTA [30,31]. CHIP carriers show higher event rates and, for TET2/JAK2 clones, causal links to atherothrombosis [34]. A unifying prognostic theme is that residual inflammatory risk—quantifiable by hsCRP—remains a powerful, independent determinant of recurrent events and mortality across all these conditions [6,7,92].

## 10. Therapeutic Interventions to Reduce the Impact of Accelerated Atherosclerosis

AA requires prompt, intensive management to reduce long-term cardiovascular risk. The principal goals are to halt or slow plaque progression and reduce MACE and other clinical complications [97]. Management combines general measures targeting modifiable traditional and non-traditional risk factors with patient-tailored strategies for specific conditions (FH, RA, SLE, high genetic risk—polygenic risk, and other situations) [98,99].

The treatment of AA is governed by three major principles:

(1) Rapid and aggressive action: Since the pathology is rapidly progressing and the clinical complications can even be in the sphere of MACE, the slow adjustment and “wait and see” approach is not the way. An aggressive treatment with a steep curve of risk reduction, both for short and long term, is the norm [98,100].
(2) Comprehensive treatment: General maximal measures of atherosclerosis management, combined with a personalized medicine approach for patients presenting specific disorders (FH, high Lp(a) levels, rheumatic disorders, autoimmune disorders, or high polygenic risk).
(3) Periodic clinical and biochemical reassessment, with non-invasive imaging used selectively when it is expected to refine risk or alter management: Invasive coronary angiography should be reserved for standard clinical indications rather than routine AA surveillance [45,97].

Drugs sometimes have pleiotropic effects: they target primarily a causal point/process, but they have additive smaller local and systemic effects through other pathways (e.g., a statin changes the endothelial signalling, the redox state and behaviour/signalling of cells in the vascular wall).

Since AA has consequences of extreme importance, this chapter will present established avenues and innovative treatments—a rapidly progressing field with major implications.

### 10.1. Diet

Lifestyle interventions are tremendously important, since many modifiable nontraditional risk factors are represented by obesity, physical inactivity/sedentary lifestyle, diet, and alcohol intake. Although some patients may initially view lifestyle advice as unrelated to medical treatment, effective communication and education can support active participation in care. With correct guidance, they will be able to take the necessary steps to follow the lifestyle interventions and also follow the medical treatment for a long period of time [101]. Physical activity contributes to a reduction in atheroma volume [102].

### 10.2. Traditional Risk Factor Mitigation

#### 10.2.1. Diabetes Mellitus Control

Diabetes mellitus is a major determinant of atherosclerotic risk and requires strict metabolic control in close collaboration with diabetes specialists.

A CCTA-driven therapeutic strategy in type 2 diabetic patients did not demonstrate superiority to the classic strategy; moreover, the rates of events in the two groups were very low for ischemic major cardiovascular events (4.4% (20 events) vs. 3.8% (17 events); hazard ratio, 1.15 (95% CI, 0.60–2.19); *p* = 0.68 [103]).

In diabetic patients, comparing patients with >2RF (risk factors) with those <1RF by performing dipyridamole myocardial contrast echocardiography resulted in similar values of approximately 60% for positive ischemic results, as confirmed by coronary angiography 64.6% vs. 65.5%, *p* = 0.92. The difference consisted in the complexity of coronary anatomy, with higher prevalence of three-vessel disease, severe occlusion (31.2% vs. 3.8%, *p* < 0.001) and diffuse disease >2RF vs. <1RF [104].

#### 10.2.2. Smoking

Smoking is a traditional cardiovascular risk factor with dose-dependent effects; its pro-atherosclerotic impacts may vary according to individual genetic and transcriptional profiles [105,106]. The best outcome is obtained by smoking cessation. However, new studies present that nicotine per se is a cardiovascularly active drug with a pro-atherosclerotic effect manifested through many signalling pathways, including signalling through acetylcholine receptors in the endothelium and macrophages, catecholamine, and pro-inflammatory activating intracellular pathways. As such, the use of alternative nicotine products should also be discontinued; they should be used just for short periods to help smoking cessation [107]. Serial IVUS studies reported PAV progressions of 0.8–1.5% in smokers compared with 0.2–0.4% in non-smokers [108].

#### 10.2.3. Blood Pressure

Hypertension is a major contributor to atherosclerosis through a plethora of mechanisms. With hypertension treatment guidelines in place, the task may seem simple, but the specialist must apply a personal expert opinion to customize the treatment regimen and provide the optimal combination for the patient’s specific needs and risk constellation. Antihypertensive drug classes may differ in their metabolic and vascular effects, particularly in patients with AA. Choosing the best combination of drugs can pose a difficult challenge when integrating all the patient’s needs [109].

Diuretics alone can prove to be a difficult choice. Thiazides have been demonstrated to induce modifications in lipid metabolism leading to increases in cholesterol and TG levels [110]. Loop diuretics, although better regarding the impact on lipid metabolism, with smaller increases in cholesterol and TG levels, cause, on the other hand, an insulin resistance of skeletal muscle and a poorer glucose transport and intracellular utilization; hence, a modified glucose tolerance is an important aspect for diabetic patients that requires therapy adjustment [111]. Angiotensin receptor blockers (ARBs), when used at diuretic concentrations, manifest lipid and glucose metabolic interactions, but with a smaller amplitude. These effects should be counterbalanced by specific medication and periodic biochemical monitoring.

Other hypertensive drug classes exert complex secondary effects on atherosclerosis (through cell signalling, anti-inflammatory effect, insulin resistance, oxidation state, et cetera) and their use should be carefully considered in order to choose the best regimen adapted to the specific needs of the AA patient [109].

### 10.3. Medication

#### 10.3.1. Lipid Lowering Therapies

##### Lowering Cholesterol Production

High-intensity statin regimen: Statins are a first-line medication for LDL cholesterol control. Statins represent a vast class of drugs that inhibit HMG-CoA reductase, hence reducing LDL-C [112]. They demonstrate plaque stabilization through pleiotropic effects. The accumulated evidence for primary and secondary prevention, reducing MACE, and in some cases, plaque reduction, makes them the most important candidate for treating AA patients. High-intensity treatment should generally be initiated early [100]. Statins’ effects on coronary plaque progression were discussed in the imaging section.

Bempedoic acid is a molecule that lowers cholesterol synthesis by inhibition of adenosine triphosphate-citrate lyase, a hepatocyte enzyme that acts upstream of HMG-CoA reductase. It can be used in cases of statin intolerance, exhibiting fewer adverse effects, such as lower levels of muscle-related symptoms (CLEAR clinical trial outcomes) [113]. It can be prescribed in combination with ezetimibe. There are no large trials testing PAV reduction.

##### Agents Limiting Cholesterol Uptake

Fibrates act primarily through peroxisome proliferator-activated receptor alpha (PPAR-α)-mediated effects on triglyceride-rich lipoprotein metabolism rather than by reducing intestinal cholesterol uptake [114]. Ezetimibe acts as an inhibitor of the Niemann-Pick C1 intracellular uptake of cholesterol at the enterocyte and hepatocyte levels. Usually, it is used as second-line medication, but in the case of AA patients, early addition of ezetimibe may facilitate rapid achievement and maintenance of LDL-C targets [115]. Positive results were obtained by combining ezetimibe with a statin in the PRECISE-IVUS trial. This effect was noticed in other combined therapies with ezetimibe, which on average doubled the rate of plaque atheroma volume reduction [116]. Measurable plaque regression was obtained in 78% of exposed patients [117].

As to the long-term perspective, imaging data are lacking and are very difficult to obtain in a comparable manner. In IMPROVE-IT, ezetimibe added to simvastatin produced a 6.4% relative reduction in the primary cardiovascular endpoint over a median follow-up of approximately 6 years [115]; the trial did not directly assess plaque volume. A meta-analysis of pooling 7407 patients evaluated with serial IVUS showed a 19–25% reduction for every 1% absolute decrease in atheroma volume [118].

##### LDL Receptor-Targeted Approach

This therapeutic approach aims primarily to reduce the activity or production of PCSK9. LDL-R can be recirculated to the cell surface from endosomal vesicles because its binding with LDL is reversible. PCSK9 binds LDL receptors and directs them from endosomes to lysosomal degradation, thereby reducing receptor recycling. The advent of PCSK9 inhibitors opened a new path for reducing the overall risk in post-MI patients. Two innovative directions are used for PCSK9 modulation: monoclonal antibodies and genetic silencing of transduction for the protein.

Monoclonal antibodies can serve as a novel class of lipid-lowering drugs. Alirocumab is a PCSK9 monoclonal antibody first approved by Food and Drug Administration (FDA) in 2015 as a second-line treatment option for better LDL cholesterol control in patients with poor results to statin or with statin intolerance. In 2021, the FDA expanded the indication for homozygous FH [119]. Clinical trials have also evaluated alirocumab in paediatric patients with familial hypercholesterolemia [120]. More insight was obtained from IVUS, optical coherence tomography and near-infrared spectroscopy intracoronary scans in non-culprit but diseased coronary vessels, after 52 weeks of alirocumab over the maximum tolerated doses of statins. In the study population, a significant regression in atherosclerosis was obtained, as expressed through PAV regression [119]. In a post hoc analysis of the same PACMAN AMI trial, it was proven that the highest therapeutic yield was obtained in those without very high-risk factors [121]. Alirocumab and evolocumab have established regulatory indications for LDL-C lowering in appropriate patients with ASCVD and familial hypercholesterolaemia; their use was also relevant in the context of the ODYSSEY OUTCOMES and FOURIER studies, which also demonstrated cardiovascular-event reduction [45,122,123].

Inclisiran is a small interfering RNA (siRNA) novel drug that targets and inhibits the translation of PCSK9 mRNA. By lowering the PCSK9 proprotein convertase level, it produces a decrease in LDL cholesterol. It has received approval for treatment of patients with ASCVD and FH in many regions of the EU (2020), the UK (2021), the USA (2021), and China (2023). It is an approved LDL-C-lowering therapy in multiple jurisdictions [124]. Definitive cardiovascular-outcomes evidence remains pending, and its role in AA specifically is therefore extrapolated rather than established [45].

##### Agents for Angiopoietin-like 3 Modulation

ANGPTL3 and ANGPTL4 are secreted angiopoietin-like proteins, not cell-membrane receptors; they regulate lipoprotein and endothelial lipase activity and thereby influence lipid metabolism. This group is also represented by two classes of innovative drugs: monoclonal antibodies and SiRNA. Because this pathway is distinct from LDL-receptor modulation, ANGPTL3 inhibition may provide additive lipid-lowering effects and may provide additional lipid lowering in appropriately selected patients, but this should not be interpreted as an AA-specific indication [125].

Evinacumab is a monoclonal antibody directed against ANGPTL3, approved as adjunctive LDL-C-lowering therapy for HoFH; age eligibility is jurisdiction-specific [126]. Binding the ANGPTL3 results in enhanced activity of endothelial lipase and lipoprotein lipase, thus reducing LDL-C levels by up to 47%. Its indication is HoFH, and it should not be presented as a proven AA-specific therapy [126,127]. SHR-1918 is another fully human ANGPTL3 monoclonal antibody with phase 2 HoFH data showing marked LDL-C reduction; later-stage development is ongoing, but cardiovascular-outcome benefit and an AA-specific role remain unproven [128].

Olezarsen is a GalNAc-conjugated antisense oligonucleotide targeting APOC3 mRNA; it is not an siRNA. It lowers triglycerides and has been approved in the United States and European Union as an adjunct to diet for adults with familial chylomicronaemia syndrome (FCS) [129,130]. Its licensed indication is FCS, and evidence from hypertriglyceridaemia studies should not be extrapolated to recommend olezarsen when AA is merely suspected [131].

Several additional ANGPTL3-directed RNA therapies are in development. Zodasiran is an RNA-interference therapy currently being evaluated in the phase 3 YOSEMITE trial in adolescents and adults with HoFH [132]. Solbinsiran is a GalNAc-conjugated siRNA targeting ANGPTL3; the PROLONG-ANG3 randomized phase 2 trial demonstrated durable reductions in triglycerides and other atherogenic lipoproteins in mixed dyslipidaemia [133]. Neither programme has established cardiovascular-outcome benefit or a specific therapeutic role in AA.

##### Lipoprotein(a) Lowering Agents

Several Lp(a)-targeted therapies are in clinical development. Their biological efficacy in lowering Lp(a) is substantial, but clinical benefit must be established in cardiovascular-outcomes trials before these agents can be considered standard therapy for ASCVD or AA. As of September 2026, no targeted Lp(a)-lowering therapy has regulatory approval for cardiovascular-risk reduction [134].

Zerlasiran is an siRNA targeting LPA with randomized phase 2 data demonstrating large and sustained reductions in Lp(a) [135]. Olpasiran is another LPA-targeted siRNA; the phase 3 OCEAN(a)-Outcomes trial is evaluating whether Lp(a) lowering reduces major coronary events in patients with established ASCVD and elevated Lp(a) [136]. Neither agent should be described as an established treatment for atherosclerosis before outcomes and regulatory data are available.

Pelacarsen is a hepatocyte-directed antisense oligonucleotide targeting apo(a) synthesis. The phase 3 Lp(a)HORIZON cardiovascular-outcomes trial tested whether lowering Lp(a) reduces major cardiovascular events in patients with established cardiovascular disease and elevated Lp(a) [137]. In September 2026, topline results indicated that pelacarsen lowered Lp(a) but did not reduce the prespecified composite cardiovascular endpoint compared with placebo [134]. This negative outcome emphasizes that biomarker lowering cannot be assumed to translate into clinical benefit.

Muvalaplin is an oral small-molecule inhibitor of Lp(a) assembly that disrupts the interaction between apo(a) and apoB100, thereby lowering circulating Lp(a); it is not primarily an LDL-C-lowering drug [138]. The phase 3 MOVE-Lp(a) outcomes trial is evaluating whether this reduction translates into fewer major cardiovascular events [139]. Until outcome data and regulatory review are available, muvalaplin remains investigational.

##### CETP Inhibitors

Obicetrapib is an oral selective cholesteryl ester transfer protein (CETP) inhibitor. Phase 3 BROADWAY and TANDEM have reported substantial LDL-C lowering, including with the obicetrapib–ezetimibe fixed-dose combination [140,141]. In July 2026, the EMA Committee for Medicinal Products for Human Use adopted a positive opinion recommending marketing authorization for Ubeslo (obicetrapib), but a positive CHMP opinion should not be described as final European Commission authorization [142]. Obicetrapib should be presented as an LDL-C-lowering therapy under regulatory review rather than as a treatment proven specifically for AA.

##### Gene Editing

In vivo gene editing is an investigational strategy intended to produce durable modification of lipid-related targets such as PCSK9, ANGPTL3, or LPA. Although CRISPR-based therapies are approved for selected haematological diseases, this does not establish cardiovascular gene editing as safe or effective. In primates, permanent PCSK9 editing has produced sustained lipid lowering [143]. More recently, the phase 1 CTX310 programme targeting hepatic ANGPTL3 demonstrated dose-dependent reductions in ANGPTL3 and atherogenic lipids, with one-year follow-up supporting persistence of the effect [144,145]. Such studies remain early-phase; long-term safety, off-target effects, reversibility, and cardiovascular-outcome benefit require further evaluation. These approaches should therefore be described as investigational rather than ‘thrilling opportunities’ for AA.

### 10.4. Personalised Approach

Patients with identifiable genetic conditions, persistent inflammation, or autoimmune/rheumatic disease could benefit more from a personalized add-on therapy.

#### 10.4.1. Dyslipidemia with Genetic Determinants

Dyslipidemia with genetic determinants will benefit from adjusted therapy. For patients who present FH alongside, a combined maximal regimen of ezetimibe, statin, PCSK9 inhibitor and plasma LDL apheresis might be necessary. For patients with high polygenic risk, evaluation in accordance with clinical findings can offer the rationale for early and aggressive therapy initiation.

#### 10.4.2. High and Persistent Inflammatory Status

Low-dose colchicine reduced ischemic cardiovascular events in COLCOT after recent myocardial infarction and in LoDoCo2 among patients with chronic coronary disease [146,147]. In contrast, CLEAR SYNERGY found no reduction in major cardiovascular events when colchicine was initiated early after acute myocardial infarction, despite lowering inflammatory markers [148,149]. These differences underscore the importance of population selection, timing, background therapy, safety, renal and hepatic function, and clinically relevant drug interactions, including CYP3A4/P-glycoprotein interactions. None of these trials specifically enrolled patients defined by accelerated plaque progression; colchicine should therefore not be presented as an AA-specific therapy.

In another clinical trial (CANTOS), canakinumab, an anti-IL1β monoclonal antibody, reduced MACE for patients with high CRP and prior MI [150]. It is notable that the potent immune suppression comes with a high infection risk. Monoclonal antibodies against IL6 and TNFα are also investigated, with promising results. The IL-6 ligand antibody ziltivekimab reduced hsCRP by approximately 90% in phase 2 (RESCUE) and is being tested for events in the ZEUS trial, which randomized 6376 participants with atherosclerotic disease, CKD, and hsCRP ≥ 2 mg/L—a population for whom colchicine is often contraindicated [6,93]. A new class of agents in the early stages of research are the inflammasome inhibitors. These anti-inflammatory trials were conducted in defined ASCVD or high-inflammatory-risk populations rather than cohorts selected for accelerated plaque progression, so their results should not be presented as direct evidence for an AA-specific treatment strategy.

#### 10.4.3. Autoimmune and Rheumatic Disease

Autoimmune and rheumatic disease control through aggressive treatment conducted by the respective specialists should be envisaged.

### 10.5. Revascularization

In AA, high-risk anatomy patients or those with refractory ischemia should be subjected to percutaneous coronary intervention or coronary artery bypass grafting without delay.

## 11. Conclusions

AA is best understood as conventional atherogenesis amplified by disease-specific inflammation and, in some settings, distinct calcific arteriosclerosis. Its defining clinical problem is potential under-prediction by standard risk tools; selected imaging can refine risk, whereas PCAT attenuation, IL-6, GDF-15, and circulating miRNA panels remain investigational for routine AA management. Genetic determinants, notably CHIP and Lp(a), refine both risk stratification and treatment selection. Therapeutically, foundational LDL lowering and rigorous disease control should be distinguished from anti-inflammatory and emerging molecular strategies whose evidence comes from specific ASCVD populations rather than AA-defined cohorts. Residual inflammatory risk may contribute importantly in selected populations, but it should not be regarded as the sole remaining therapeutic target.

We need a change of paradigm to assist in assessing the real cardiovascular risk and more thorough approaches in monitoring and tailoring adequate therapies for these patients.

## Figures and Tables

**Table 1:** Clinical conditions associated with accelerated or disproportionate atherosclerotic disease: mechanisms, evidence, and current clinical implications.

Condition	Dominant Accelerator(s)	Evidence/Interaction with Atherogenesis	Current Clinical Implication	References
Rheumatoid arthritis	Systemic inflammation; endothelial activation; altered lipoprotein function; disease activity and glucocorticoid exposure	Higher event risk and increased subclinical plaque burden/progression; inflammation amplifies apoB-driven atherogenesis	Aggressive conventional risk-factor control plus effective disease control	[1]
Systemic lupus erythematosus	Chronic immune activation; autoantibodies; endothelial dysfunction; glucocorticoid exposure	Premature events and prospective plaque progression; traditional and disease-specific factors interact	Conventional prevention plus sustained control of disease activity	[2,9,10]
Antiphospholipid syndrome	Thrombo-inflammation; endothelial activation; antiphospholipid antibodies	Increased carotid/femoral plaque burden in prospective cohorts; atherogenesis coexists with thrombosis	Manage thrombosis according to APS indications and optimize ASCVD risk factors	[11,12]
Chronic kidney disease	Uraemia; mineral-bone disorder; oxidative stress; inflammation; loss of calcification inhibitors	Rapid CAC progression and high event rates; intimal atheroma coexists with medial calcification	Guideline-directed CKD and ASCVD risk management; distinguish atheroma from medial calcification	[5,13,14]
HIV	Residual immune activation and inflammation plus traditional/metabolic risk factors	Excess cardiovascular risk; statin benefit demonstrated in REPRIEVE	Assess and treat conventional risk factors; statin therapy when indicated	[15]
Diabetes mellitus	Hyperglycaemia; insulin resistance; oxidative stress; endothelial dysfunction	Higher plaque burden and event risk; accelerates canonical lipid-driven atherogenesis	Intensive multifactorial diabetes and ASCVD risk management	[16]
Psoriasis/psoriatic disease	Systemic inflammatory signalling and vascular inflammation	Higher prevalence of subclinical and high-risk coronary plaque	Conventional risk-factor control plus effective inflammatory-disease treatment	[17,18]
Cardiac allograft vasculopathy	Alloimmune and innate immune injury; endothelial dysfunction; non-immune risk factors	Recognized accelerated coronary vasculopathy; IVUS/OCT can detect early diffuse intimal disease	Dedicated transplant surveillance and immunosuppression/ASCVD management	[19,20]

APS, antiphospholipid syndrome; ASCVD, atherosclerotic cardiovascular disease; CAC, coronary artery calcium; CKD, chronic kidney disease; HIV, human immunodeficiency virus; IVUS, intravascular ultrasound; OCT, optical coherence tomography; REPRIEVE, Randomized Trial to Prevent Vascular Events in HIV.

## Data Availability

No new data were created or analyzed in this study. Data sharing is not applicable to this article.

## Abbreviations

The following abbreviations are used in this manuscript:

AA	Accelerated atherosclerosis
ABC	ATP- binding cassette
ANGPTL3	Angiopoietin-like protein 3
ASO	Antisense oligonucleotide
ASCVD	Atherosclerotic cardiovascular disease
APOB	Apolipoprotein B
APOC3	Apolipoprotein C-III
ARBs	Angiotensin receptor blockers
ATP	Adenosine triphosphate
AUC	Area under the curve
CAC	Coronary artery calcium
CAD	Coronary artery disease
CAV	Cardiac allograft vasculopathy
CCTA	Coronary computed tomography angiography
CDKN2A/2B	Cyclin-dependent kinase inhibitor 2A/B
CDKN2B-AS1	Cyclin-dependent kinase inhibitor 2B antisense RNA 1
CHIP	Clonal hematopoiesis of indeterminate potential
CIMT	Carotid intima–media thickness
CKD	Chronic kidney disease
COX2	Cyclooxygenase-2
CRIC	Chronic renal insufficiency cohort
CRISPR	Clustered regularly interspaced short palindromic repeats
CETP	Cholesteryl ester transfer protein
CXCL12	C-X-C motif chemokine ligand 12
CT	Computed tomography
DNA	Deoxyribonucleic acid
DNMT3A	DNA cytosine-5-methyltransferase 3A
EMA	European Medicines Agency
FCS	Familial chylomicronaemia syndrome
FDA	Food and Drug Administration
FH	Familial hypercholesterolemia
GDF-15	Growth differentiation factor-15
GWAS	Genome-wide association studies
HDAC	Histone deacetylases
HDL	High-density lipoprotein
HIV	Human immunodeficiency virus
HMG-CoA	3-hidroxi-3-metilglutaril-coenzyme A
hsCRP	High-sensitivity C-reactive protein
IL	Interleukin
IMT	Intima–media thickness
IVUS	Intravascular ultrasound
JAK2	Janus kinase 2
LINC00493	Long intergenic non-protein-coding RNA 493
LDL	Low-density lipoprotein
Lp(a)	Lipoprotein(a)
MACE	Major adverse cardiovascular events
MALAT1	Metastasis-associated lung adenocarcinoma transcript 1
MI	Myocardial infarction
NCPV	Non-calcified plaque volume
NF-κB	Nuclear factor-κB
OCT	Optical coherence tomography
PCATa	Pericoronary adipose tissue attenuation
PAV	Percent atheroma volume
PCSK9	Pro-protein convertase subtilisin/kexin type 9
PPAR-α	Peroxisome proliferator-activated receptor alpha
PRS	Polygenic risk scores
QTL	Quantitative trait locus
RA	Rheumatoid arthritis
RF	Risk factors
RISC	RNA-induced silencing complex
RNA	Ribonucleic acid
SENCR	Smooth muscle- and endothelial cell-enriched migration/differentiation-associated long non-coding RNA
siRNA	Small interfering RNA
SLE	Systemic lupus erythematosus
SMILR	Smooth muscle-induced long non-coding RNA, enhancer of proliferation
SORT1	Sortilin1
TAV	Total atheroma volume
TET2	TET methylcitosine dioxygenase 2
TG	Triglyceride
